# Pregnancy-associated blistering in a patient with junctional epidermolysis bullosa

**DOI:** 10.1016/j.jdcr.2024.01.026

**Published:** 2024-02-05

**Authors:** Lily Hertel, Mollie Hutton, Paul Bogner, Raminder Grover, Drew Kuraitis

**Affiliations:** a DeBusk College of Osteopathic Medicine, Lincoln Memorial University, Harrogate, Tennessee; b Department of Dermatology, Roswell Park Comprehensive Cancer Center, Buffalo, New York; c Clinical Genetics Service, Roswell Park Comprehensive Cancer Center, Buffalo, New York; d Beutner Laboratories, Buffalo, New York; e Department of Dermatology, Tulane University, New Orleans, Louisiana

**Keywords:** dermatosis of pregnancy, epidermolysis bullosa, gestational pemphigoid, junctional epidermolysis bullosa, pemphigoid gestationis, polymorphic eruption of pregnancy, pregnancy

## Introduction

Epidermolysis bullosa (EB) is a group of rare genetic disorders that manifest as blistering and fragility of the skin after minor trauma.^1^ Junctional EB (JEB) is an autosomal recessive form of EB which is characterized by skin blistering with cleavage through the lamina lucida. Severe forms of JEB can be fatal in infancy, but less severe, or generalized-intermediate forms, may have a normal life expectancy. Polymorphic eruption of pregnancy (PEP), formerly known as pruritic urticarial papules and plaques of pregnancy, is a third trimester dermatosis of pregnancy characterized by pruritic urticarial papules and plaques, without significant association of fetal harm.^2^ Pemphigoid gestationis (PG) is a rare blistering dermatosis of pregnancy that occurs in the second or third trimester. PG typically presents with eruptive pruritic papules, plaques and bullae on the trunk and is associated with prematurity.^2^ Although PEP is more common (1 in 160 pregnancies) than PG (1 in 50,000 pregnancies),^2^ neither have been reported in patients with JEB.

## Case presentation

**Fig 1:**
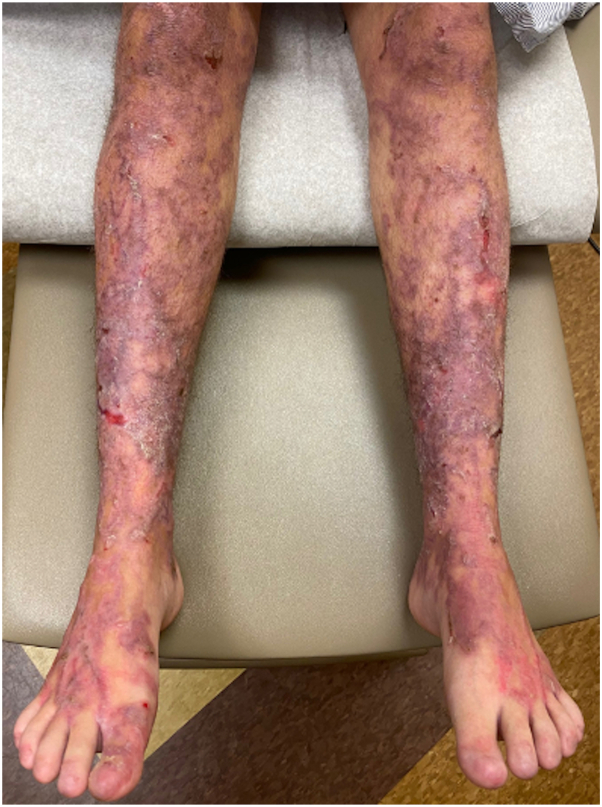
Initial patient presentation with brightly erythematous eroded plaques and blisters to the lower portion of the legs, as well as toenail dystrophy.

**Fig 2:**
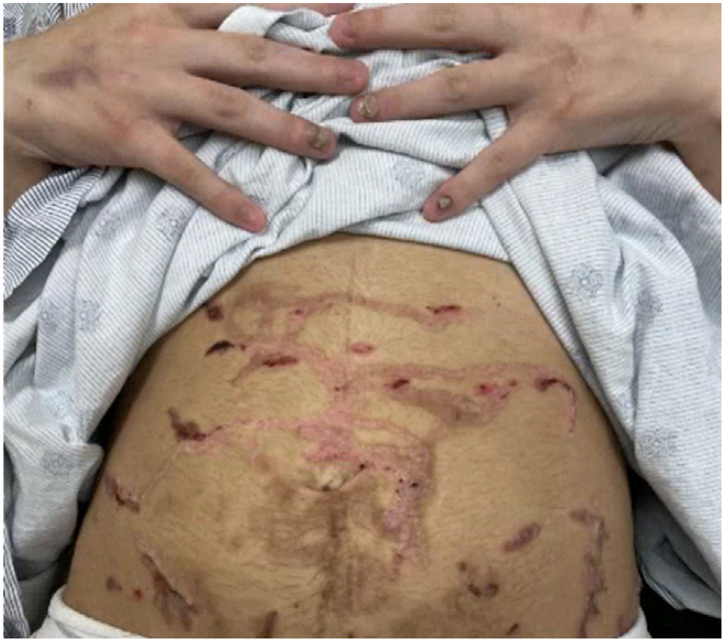
Linear, eroded plaques and bullae across the abdomen at sites of recent traumatic excoriation.

**Fig 3:**
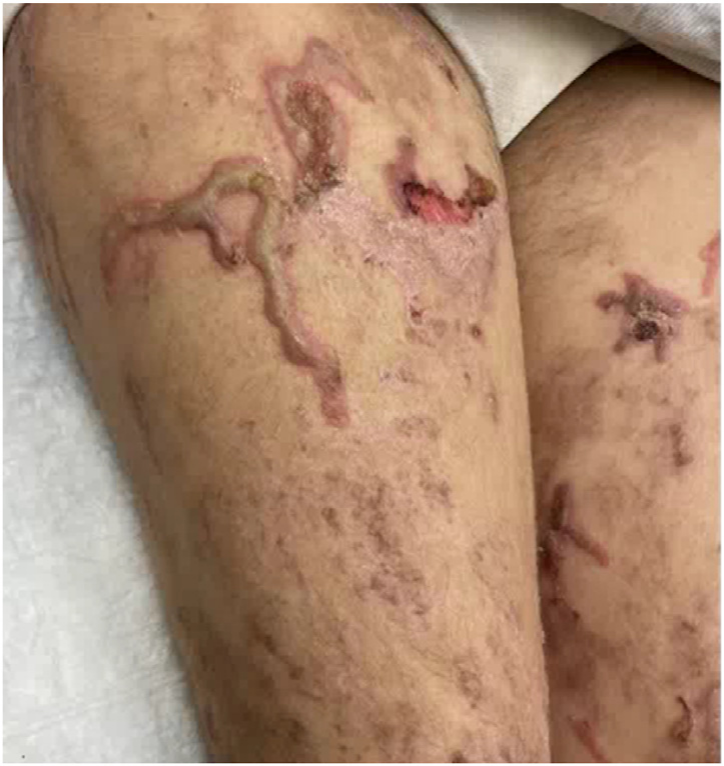
Intact erythematous linear bullous plaques, shallow erosions, and background ill-defined erythema to the thigh.

**Fig 4:**
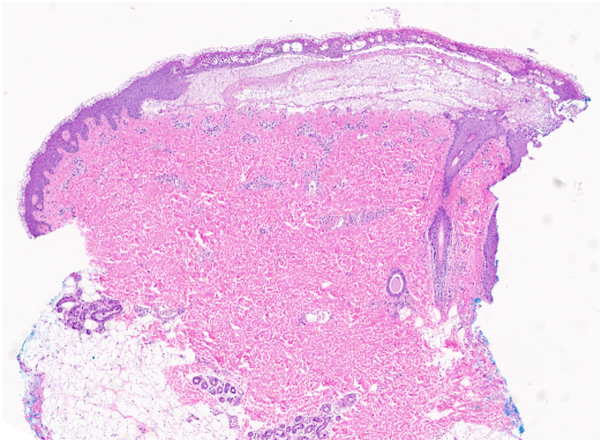
Histopathology of intact blister demonstrating subepidermal vesiculation with a mixed inflammatory infiltrate, including eosinophils within the blister space and in the underling dermis. (Hematoxylin-eosin stain; original magnification: ×16.)

A 21-year-old primigravid woman with a known history of EB presented at 32 weeks’ gestation with painful blisters and itching on the trunk and extremities. Her EB presented in infancy, characterized by fragile skin and recurrent blistering, and had typically been restricted to the distal extremities, accompanied by itching at times. Intense itching developed initially to the thighs, then to the abdomen and distal extremities toward the end of her second trimester. This intense itching was accompanied by formation of blisters that were pruritic and larger than her typical EB blisters. On examination, she had eroded erythematous plaques and bullae on the lower portion of the legs and scar-like plaques (Fig 1), with background erythema and excoriations. Many fingernails and toenails were absent. The differential diagnosis included PEP, PG and intrahepatic cholestasis of pregnancy, and biopsies were deferred at that time by the patient. She completed a 10-day course of prednisone (maximum dose of 20 mg), which resolved pruritus and blistering for at least 3 weeks. Pruritus gradually recurred shortly before giving birth and intensified immediately postpartum. Formation of large blisters reportedly recurred within 2 weeks before giving birth at 38 weeks’ gestation via spontaneous vaginal delivery to a healthy baby and continued into the postpartum course. The patient represented 10 days postpartum, and physical examination was notable for linear eroded plaques and intact linear bullae across the abdomen (Fig 2) and thighs (Fig 3). Biopsies were performed, and she started a prednisone taper, with a starting dose of 30 mg. Biopsy of an intact blister edge showed subepidermal vesiculation with a mixed inflammatory infiltrate, including eosinophils within the blister space and throughout the underlying dermis (Fig 4). Both periodic acid–Schiff and collagen IV staining localized to the blister floor. Biopsy of perilesional erythema demonstrated a sparse perivascular infiltrate with eosinophils. Direct immunofluorescence (DIF) did not demonstrate any immune deposits in the dermoepidermal junction. Indirect immunofluorescence (IIF) tests were negative for IgG or IgG4 basement membrane zone antibodies on monkey esophagus substrate, and both anti-BP180 and anti-BP230 were negative by enzyme-linked immunosorbent assay (ELISA). The localizations of the antigens of type VII collagen, laminin 332, type XVII collagen, plectin, collagen IV, and keratin 14 by immunofluorescence mapping studies was suggestive of JEB. The reactions for laminin 332 and α6 integrin were weak compared with the normal-appearing skin controls. Molecular genetic testing was completed through a 27-gene EB panel, identifying a likely pathogenic mutation in the LAMC2 gene (c.497_501dup), as well as a variant of uncertain significance in both the LAMC2 gene (c.2220+6 T>C) and DSP gene (c.7396 G>A). A clinical diagnosis of PEP occurring in the setting of JEB was made. After her postpartum course of prednisone, her JEB returned to its baseline, with ongoing blister formation to the distal extremities.

## Discussion

PEP and PG are dermatoses of pregnancy which may have similar dermatologic manifestations.^2^ Both PEP and PG are characterized by pruritic papules and plaques typically involving the abdomen. PEP usually presents in the third trimester, whereas PG can occur in the second or third trimester. Although both PG and PEP may present similarly, accurate diagnosis is essential as PG has greater risk of fetal morbidity,^3^ whereas PEP is not associated with fetal or maternal morbidity.^4^ This patient with JEB presented with intense pruritus and blistering in her third trimester. In her case, periumbilical involvement and bullae may favor the diagnosis of PG over PEP, but primigravid status and presence of lesions within striae would favor PEP.^2^ Intrahepatic cholestasis of pregnancy was not high on the differential diagnosis given her clinical course. Although total bile acids were not checked, parameters on a standard complete metabolic panel were unremarkable. The gold standard for diagnosis of PG is DIF findings. The presence of linear deposition of C3 with or without IgG along the dermoepidermal junction on DIF of perilesional skin is indicative of PG. Our patient’s ELISA, DIF and IIF findings were unremarkable. Bullous pemphigoid can develop rarely in patients with EB, and can show findings consistent with pemphigoid, including positive anti-BP180.^5^ The presence of anti-BP180 is highly sensitive and specific for PG^6^ and our patient’s anti-BP180 was negative. Together, the IIF, DIF, and ELISA studies do not support a diagnosis of PG in this patient, or another form of autoimmune bullous disease.

Clinically, it was suspected that the patient was experiencing PEP and that scratching led to linear bullae and erosions on the abdomen and legs. Histopathologic findings were supportive of inflamed JEB bullae, subepidermal vesiculation containing fibrin and inflammatory cells including eosinophils within the blister space and throughout the underlying dermis. Furthermore, histopathologic evaluation of perilesional, nonbullous skin demonstrated a nonspecific perivascular infiltrate with eosinophils, which may be representative of PEP. Histopathology of PEP may have varied findings, but typically includes a perivascular infiltrate with variable amounts of eosinophils.^7^ PEP is more often seen toward the end of the third trimester but may present earlier.^2^ PEP also does not usually recur after treatment, as was observed in our case. Triggers, such as skin stretching, may be involved in the pathogenesis of PEP and given our patient’s unique baseline cutaneous pathology, it is possible that underlying or propagating triggers for PEP or a PEP-like eruption could be etiologic.

Although 2 variants were identified in the patient’s LAMC2 gene during genetic testing, one of these variants is currently classified as a variant of uncertain significance, meaning it is unclear at this time whether this variant impacts gene function. Additionally, it is unclear whether the 2 identified LAMC2 variants exist in *cis* or *trans*. Different explanations for why 2 pathogenic mutations were not identified include the following: (1) the identified LAMC2 variant (c.2220+6 T>C) may actually be pathogenic, but adequate information is not yet available to make this interpretation, (2) a different pathogenic mutation in the LAMC2 gene may still exist but was not detected because of the limits of technology, (3) the patient’s clinical diagnosis of JEB may be because of mutations in an as yet unidentified or untested gene. Despite the discordant molecular result, and the patient has been diagnosed with JEB and appears to have an intermediate form.

Although PEP is more frequent than PG, it is important to consider PG in a pregnant woman presenting with a bullous eruption because of impact on fetal morbidity. This case demonstrates an unusual coexistence of a PEP-like eruption superimposed on JEB, presenting as a pruritic bullous eruption in the third trimester.

## Conflicts of interest

None disclosed.
